# Trained innate immunity as a potential link between preeclampsia and future cardiovascular disease

**DOI:** 10.3389/fendo.2024.1500772

**Published:** 2024-12-16

**Authors:** Ivo Carrasco-Wong, Javiera M. Sanchez, Jaime A. Gutierrez, Delia I. Chiarello

**Affiliations:** Escuela de Tecnología Médica, Facultad de Medicina y Ciencia, Universidad San Sebastián, Santiago, Chile

**Keywords:** preeclampsia, maternal cardiovascular health, long-lasting effects, trained immunity, DAMPs (damage-associated molecular pattern molecules)

## Abstract

Preeclampsia (PE) is a complex pregnancy syndrome characterized by hypertension with or without proteinuria, affecting 2–6% of pregnancies globally. PE is characterized by excessive release of damage-associated molecular patterns (DAMPs) into the maternal circulation. This DAMP-rich milieu acts on innate immune cells, inducing a proinflammatory state characterized by elevated cytokines such as IL-1β and IL-18. This proinflammatory state in the mother and placenta results in the endothelial dysfunction strongly associated with cardiovascular disorders. While the immediate maternal and fetal risks of PE are well-documented, accumulating evidence indicates that PE also confers long-term cardiovascular risks to the mother, including hypertension, coronary heart disease, stroke, and heart failure. The underlying mechanisms connecting PE to these chronic cardiovascular conditions remain unclear. This article explores the potential role of trained innate immunity (TRIM) as a mechanistic link between PE and increased long-term cardiovascular risk. We propose that the persistent exposure to DAMPs during PE may epigenetically reprogram maternal innate immune cells and their progenitors, leading to TRIM. This reprogramming enhances the inflammatory response to subsequent stimuli, potentially contributing to endothelial dysfunction and chronic inflammation that predispose women to cardiovascular diseases later in life. Understanding the role of TRIM in PE could provide novel insights into the pathophysiology of PE-related cardiovascular complications and identify potential targets for therapeutic intervention. Further research is warranted to investigate the epigenetic and metabolic alterations in innate immune cells induced by PE and to determine how these changes may influence long-term maternal cardiovascular health.

## Preeclampsia and maternal long-lasting effects

Preeclampsia (PE) is a serious syndrome of pregnancy, characterized by hypertension with or without proteinuria, which can lead to the severe condition of eclampsia (1). In patients without signs of proteinuria, the diagnosis of the syndrome considers the presence of thrombocytopenia or elevated liver transaminase levels (2). It is estimated that in general population the prevalence of preeclampsia is 2–6% (3). The syndrome is subclassified into either early-onset PE (EOPE) or late-onset PE (LOPE) (1), with the onset of pathological signs falling into <34^th^ and >34^th^ week of gestation, respectively, being EOPE the syndrome which presents the most severe additional symptoms and signs, such as proteinuria, hepatic damage or pulmonary edema, among others (1).

Increasing evidence shows that the syndrome has a long-lasting deleterious effect on their cardiovascular health. Thus, four meta-analysis made on 22 (4), 43 (5), 15 (6) and 21 studies (7) showed that in short-, medium- and long-term (*i.e.* follow-up from 1 month to 34 years (4–7)), women who had preeclampsia have increased risk of I) heart failure (adjusted risk ratio [aRR], 4.19; 95% confidence interval [CI], 2.09–8.38) (4); II) coronary heart disease (aRR, 2.50; 95% CI, 1.43–4.37) (4); III) coronary heart death (aRR, 2.10; 95% CI, 1.25–3.51) (4); IV) CV disease (aRR, 1.85; 95% CI, 0.80–4.29 (4) and odds ratio (OR), 2.28; 95% CI 1.87–2.77) (5); V) CV disease death [aRR, 2.21; 95% CI, 1.83–2.66 (4), OR, 2.89, 95% CI 1.71–4.89 (5) and RR, 2.29; 95% CI, 1.73-3.04) (6)]; VI) stroke (aRR, 1.81; 95% CI, 1.29–2.55) (4); VII) stroke death (aRR, 1.97; 95% CI, 0.80–4.88) (4); VIII) cerebrovascular disease (RR, 2.03; 95% CI, 1.54-2.67) (6); IX) peripheral arterial disease (RR, 1.87; 95% CI, 0.94-3.73) (6); and, X) hypertension [RR, 3.13, 95% CI 2.51–3.89) (5) and OR, 3.19, 95% CI, 1.52–6.70 (7)]. Thus, the effects of preeclampsia not only impact maternal health during the pregnancy but also induce subclinical alteration which can remain silent for years, increasing her cardiovascular risk. In this regard, increased microalbuminuria, a was found in association whit high hypertension risk in mothers who had PE 7 (8) and 10 (9) years before. Although, the pathophysiological mechanism of the findings is unknown, this slight alteration of the glomerular filtration is independently associated with high stroke (10) and coronary heart disease (11) risks. Nowadays, there is no knowledge about the pathophysiological mechanisms underlying the cardiovascular risk in mothers who had PE.

It has been agreed that the most likely etiology of PE is a poor remodeling of the spiral arteries and veins during early placentation. Meanwhile, LOPE appears to be linked to maternal factors, such as the inability of the cardiovascular system to meet the increasing metabolic needs of the fetoplacental unit, rather than issues with the placentation process (12). The PE condition maintains a pernicious low blood flow in a condition of high pressure (Jet-type), generating vascular mechanical stress, hypoxia (13), and syncytiotrophoblast (STB) dysfunction (14). STB is a multinucleated cell layer of fetal origin that covers the chorionic villi and is in direct contact with maternal blood (15). Thus, it has been reported that STB stress induced by the Jet-type blood flow can be characterized by: 1) STB damage markers, such as increased mitochondrial dysfunction, apoptotic markers, reticulum stress, oxidative stress, and inflammation; 2) An excessive release of microvesicles, exosomes, and cell fragments (16); and, 3) an increased release of ‘Damage-associated molecular patterns’ (DAMPs) (17). In PE, increased DAMPs activate the NOD-, LRR- and pyrin domain-containing protein 3 (NLRP3) inflammasome, which promotes IL-1β and IL-18 maturation (18).

DAMPs are host to intracellular molecules that are not usually found in cell-free form. They can activate ‘Pattern Recognition Receptors’ (PRRs) mainly in innate immune cells, which are responsible for recognizing pattern molecules of microorganisms (19). PRRs are classified into several classes, including Toll-like receptors (TLR), nucleotide-binding oligomerization domain, Leucine-rich repeats, nucleotide-binding domain leucine-rich repeat containing receptors, retinoic acid-inducible gene 1 (RIG-1) -like receptors, and the C-type lectin receptors (20). PRRs are found in various cell types including monocytes/macrophages (21), neutrophils (22), and endothelial cells (23). The innate immune cells activated by DAMPs via TLR, switch from a tolerogenic, anti-inflammatory phenotype to a cytotoxic, pro-inflammatory phenotype (24). The activation of PRRs favors the proinflammatory status by inducing the secretion of proinflammatory cytokines (25). Under conditions of hypoxia and oxidative (17), reticulum (26), and mitochondrial (27) stress, all of them found in STB stress (16), the STB increases the releasing of several DAMPs, such as HMGB1 or cell-free fetal DNA (17). In this sense, the SBT stress contribute to the maternal pro-inflammatory milieu, which includes several increment level of circulating DAMPs and cytokines (28), are part of the pathophysiological signs of the syndrome and could activate and maintain the proinflammatory profile of innate immune cells and endothelial cell dysfunction observed in the syndrome (29). Both early-onset and late-onset preeclampsia appear to share systemic and placental inflammation as a common pathophysiological feature.

## Proinflammatory status in preeclampsia

The immunological mechanisms underlying preeclampsia have been extensively explored (24, 30), highlighting the role of adaptive and innate immune pathways in the development of this condition. During the physiological pregnancy, the inflammatory profile of the maternal immune system changes, being proinflammatory during placentation. This is proposed as a requirement for an effective invasion of the placenta and remodeling of the spiral arteries (31). In the 2nd gestation trimester, the profile changes to anti-inflammatory which is the basis of tolerance toward the fetal-placental unit. At the time of delivery, the profile becomes proinflammatory contributing to labor (31). Therefore, a large part of the pregnancy takes place with strong maternal immunomodulation, which is manifested by: I) high levels of anti-inflammatory cytokines (e.g. IL-10), immunosuppressants (e.g. progesterone), suppressive (e.g. HLA-G), and tolerogenic molecules (e.g. TGF-β) (32); and by II) an increase of anti-inflammatory immune cells that including macrophages with the anti-inflammatory phenotype (M2), and a reduction ratio of helper T lymphocytes, Th1:Th2 and Th17:Tregs (33). In general, the placenta favors immunotolerance, either through the expression of human leukocyte antigen G (HLA-G) that reduces the reactivity of natural killer lymphocytes (34), or by releasing extracellular vesicles, which induce the secretion of anti-inflammatory cytokines from the macrophages that engulf them (35). Contrarily, PE is characterized by presenting a proinflammatory state in the mother and placenta (36), which includes an increment of circulating proinflammatory mediators (e.g. TNF-α and IL-6), diminishing of anti-inflammatory cytokines (e.g. IL-10) (36), decreased circulating levels of progesterone (37), decreased HLA-G expression (38), higher ratio lymphocytes Th1:Th2 and Th17:Tregs (36), and increased activation of monocytes, neutrophils, and macrophages (39). In PE, the unbalance toward the proinflammatory status is associated with endothelial activation, leading to endothelial dysfunction and high blood pressure (40).

## Cardiovascular disorders and endothelial dysfunction/activation

Preeclampsia has been associated with microvascular dysfunction, which may contribute to the increased risk of obstructive coronary artery stenosis observed in women with a history of this condition, especially when preeclampsia is associated with preterm delivery or stillbirth (41). In physiological status, the endothelium shows a balance between vasodilator and vascular constrictor molecules, such as nitric oxide and Endothelin, respectively, which helps to maintain an anti-inflammatory and antithrombotic function (42). Cardiovascular disorders are strongly associated with endothelial dysfunction (43), characterized by an alteration in endothelium-dependent vascular relaxation, oxidative stress, and the inflammatory activation of endothelial cells (44). During endothelial activation, an overexpression of proinflammatory cytokines, chemokines, and adhesion molecules has been described in endothelial cells (43). Thus, systemic inflammation is associated with vascular diseases (45) and hypertension (46). Increasing evidence indicates that immune cells are directly involved in the onset of hypertension. In IFN-γ KO mice, a murine model of hypertension (DOCA+salt model) did not show the characteristic increment of the blood pressure of the model (47), being observed only when CD8^+^ T lymphocytes from hypertensive WT mice were transferred to knock-out (KO) individuals (47). However, not only are T cells implicated in the onset of hypertension but also innate immune cells, such as dendritic cells, monocytes/macrophages, natural killer cells, and neutrophils (48). Although adaptive immune cells have an important role in the onset of cardiovascular disorders, recently, innate cells have become relevant in the phenomenon due to the persistent activation of PRRs. It is proposed that cardiovascular diseases are related to a maladaptive inflammatory response of innate cells (49). In this sense, it was reported that circulating monocytes isolated from patients with coronary artery disease have increased NLRP3 and caspase-1 expression, both related to inflammasome formation and the elevated production of the downstream cytokines, such as IL-1β and IL-18 (50). Classically, it is described that inflammasome formation triggers pyroptosis (51). However, the neutrophils in a DAMP-rich inflammatory milieu are resistant to this type of cell death, becoming a permanent producer of IL-1β (52).

## Monocytes and neutrophils and their association with cardiovascular pathologies

Meta-analysis of 27 studies evidenced that intermediate- and non-classical monocytes are increased in persons with cardiometabolic disorders and cardiovascular disease (53). On the other hand, a high neutrophil-to-lymphocyte ratio is associated with several cardiovascular conditions, as evidenced by a meta-analysis of 38 studies (54), being proposed as a biomarker of cardiovascular health. These innate immune cells are tightly involved in the magnitude of cardiovascular lesions or alterations. In the case of animals subjected to myocardial infarction and reperfusion, the depletion of neutrophils prior to the infarct showed a significant reduction in infarct size (55). During atherogenesis, neutrophils in the intima release reactive oxygen species and proteases, which alter the endothelium integrity, allowing the recruitment and extravasation of monocytes and its further differentiation to macrophages (Reviewed in (56)). The myeloperoxidase released by neutrophils oxidates LDL, enhancing the amount of oxLDL, which together with activated macrophages generates the foam cells (56). However, despite the pro-inflammatory profile of the cardiovascular event, high basal levels of pro-inflammatory cytokines precede the occurrence of cardiovascular pathologies. Thus, a meta-analysis showed that high levels of IL-6, IL-18, and TNFα increase the risk of non-fatal myocardial infarction or coronary heart disease death (57). All those cytokines can induce endothelial dysfunction (58, 59). The activated neutrophils secrete the pro-inflammatory cytokines TNFα, IL-1β and IL-12 (60), and activated monocytes secrete IL-1β, IL-6, TNFα (61). The latter suggests that chronic activation of monocytes and neutrophils can impact endothelial function increasing the risk of cardiovascular pathologies.

In the context of preeclampsia, the pro-inflammatory environment includes activated monocytes, showing increased expression of CD11b, ICAM-1, CD14, and TLR4, an overproduction of reactive-oxygen-species (ROS), and altered secretion of cytokines (62). In the same way, neutrophil concentration is increased in maternal blood (63) along with an increment of the neutrophil activation markers CD11b and CD62L (64). Considering that a proinflammatory milieu is shared characteristic between preeclampsia and cardiovascular disorder, then an alteration of the performance of the immune system could be part of the mechanisms that increase the long-lasting cardiovascular risk in women who had preeclampsia. In this sense, ‘Trained Innate Immunity’ (65) could be the mechanism since it has recently been involved as a player in the onset of cardiometabolic diseases (65, 66).

## ‘Trained Innate Immunity’ (TRIM)

TRIM is defined as ‘the long-term functional reprogramming of innate immune cells, which is evoked by exogenous or endogenous insults leading to an altered response toward a heterologous second challenge after the return to a non-activated state’ (67). TRIM can be triggered by DAMPs, including oxidized low-density lipoprotein (oxLDL) (66), lipoprotein(A) (68), catecholamines (69), aldosterone (70), heme (71), vimentin (72), uric acid (73), S100-alarmin (74) and HMGB1 (75). DAMPs can induce TRIM by signaling through PRRs, including the receptor for advanced glycation end products (RAGE) (76), CD36 (77), and the five types of PRRs (77). Even though there is no report about TRIM in preeclampsia, it is possible to propose that it could occur since several DAMP levels are increased in maternal blood (17, 78).

The effects of TRIM induction mediated by DAMPs trigger metabolic and epigenetic modification that will lead to memory. For instance, cellular metabolic changes are necessary for epigenetic reprogramming (79), including an overexpression of glycolytic enzymes after the first stimulus (80) and an increment of lactate due to higher glucose consumption (81). Fumarate and succinate are produced as intermediates in the tricarboxylic acid cycle and glycolysis, and those intermediate are increased in trained macrophages (82). Additionally, oxidative phosphorylation also in enriched in trained cells (83). The influence of cellular metabolism on epigenetic mechanisms is already known. In TRIM, the accumulation of fumarate inhibits the demethylase activity of KDM5, a lysine demethylase of histones (84).

There is no report about TRIM being induced in preeclampsia. However, in maternal preeclamptic monocytes showed basal intracellular reactive-oxygen-species and increased oxidative burst after stimulation, which is indicative of a potentiated oxidative phosphorylation (85), similar to the observation made in monocytes trained with oxLDL (83). Noteworthy, mothers with PE, exhibited an increased percentage of classical monocytes-2 (CD14^++^, CD16^-^, HLA-DR^-^) and a decreased percentage of non-classical monocytes (CD14^+^, CD16^++^) prior to delivery (86, 87). Since classical monocytes-2 are considered as pro-tolerogenic (88) while non-classical monocytes are associated with pro-inflammatory responses (88), the altered levels observed in PE-pregnancies are proposed to reflect a compensatory mechanism aimed at counterbalancing low-grade chronic inflammation (87). Interestingly, although monocyte-2, considered as monocyte-myeloid derived suppressive cell (89), mainly by its capacity of differentiate naïve CD4+ T cells to CD4+, CD25+, Foxp3+ regulatory T-cell (Treg) (90), in Psoriasis, the induced-Treg differentiated by monocytes-2 showed a deficient suppressive activity (91). The latter suggest that in preeclampsia, a similar phenomenon could be occurring, since circulatory Treg in preeclampsia shows reduced function, with reduced expression of FOXP3 and reduced IL-10 and TGF-β secretion (92). However, there is no data on the role of monocyte subpopulations in PE or their frequency during the postpartum period. Consequently, the potential permanent programming of monocyte subpopulations remains unknown.

Resident natural killer cells (NK) in decidua (dNK) is an essential cell type during the placentation due to its activity that include the induction of the remodeling of spiral arteries by the disruption of its vascular smooth muscle cells (93) and by the interaction with extravillous trophoblast cells (94) promoting its invasion activity an arterial remodeling through INF-γ and VEGFα secretion (95). Noteworthy, the dNK from multiparous mothers showed a higher response to trophoblast interaction characterized by enhanced INF-γ and VEGFα secretion, in association with an open state of chromatin of their locus, among other loci (95). Thus, it is proposed that the physiological pregnancy can promote epigenetically a tolerance to future pregnancies (95). In preeclampsia, dNK are increased in decidua but showing reduced activity (e.g. reduced INF-y secretion) (96), which impact in the spiral arteries remodeling. In this scenario, also, it is possible to propose a memory in dNK in PE, since the mothers that have a prior pregnancy with preeclampsia have the greatest relative risk (RR) of PE in a new pregnancy, with a RR of 8.4 (7.1 to 9.9, 95% CI) (97). Also, other conditions increase the risk of PE, such as chronic hypertension with a 5.1 of RR (4.0 to 6.5, 95% CI); pregestational diabetes with a RR of 3.7 (3.2 to 4.3, 95% CI); and, pre-pregnancy BMI>30 with a RR of 2.8 (2.6 to 3.1, 95% CI) (97). In all this pathologies the activity of NK is reduced (98–100). It is proposed that NK exhaustion can be produced by chronic inflammation (101) which is found in chronic hypertension (102), diabetes (103), obesity (104), and preeclampsia (described above). Then, chronic inflammation observed in several pathologies with high risk of PE may generate a pro-exhaustion memory in circulatory and decidual NKs favoring the onset of PE.

**Figure 1:**
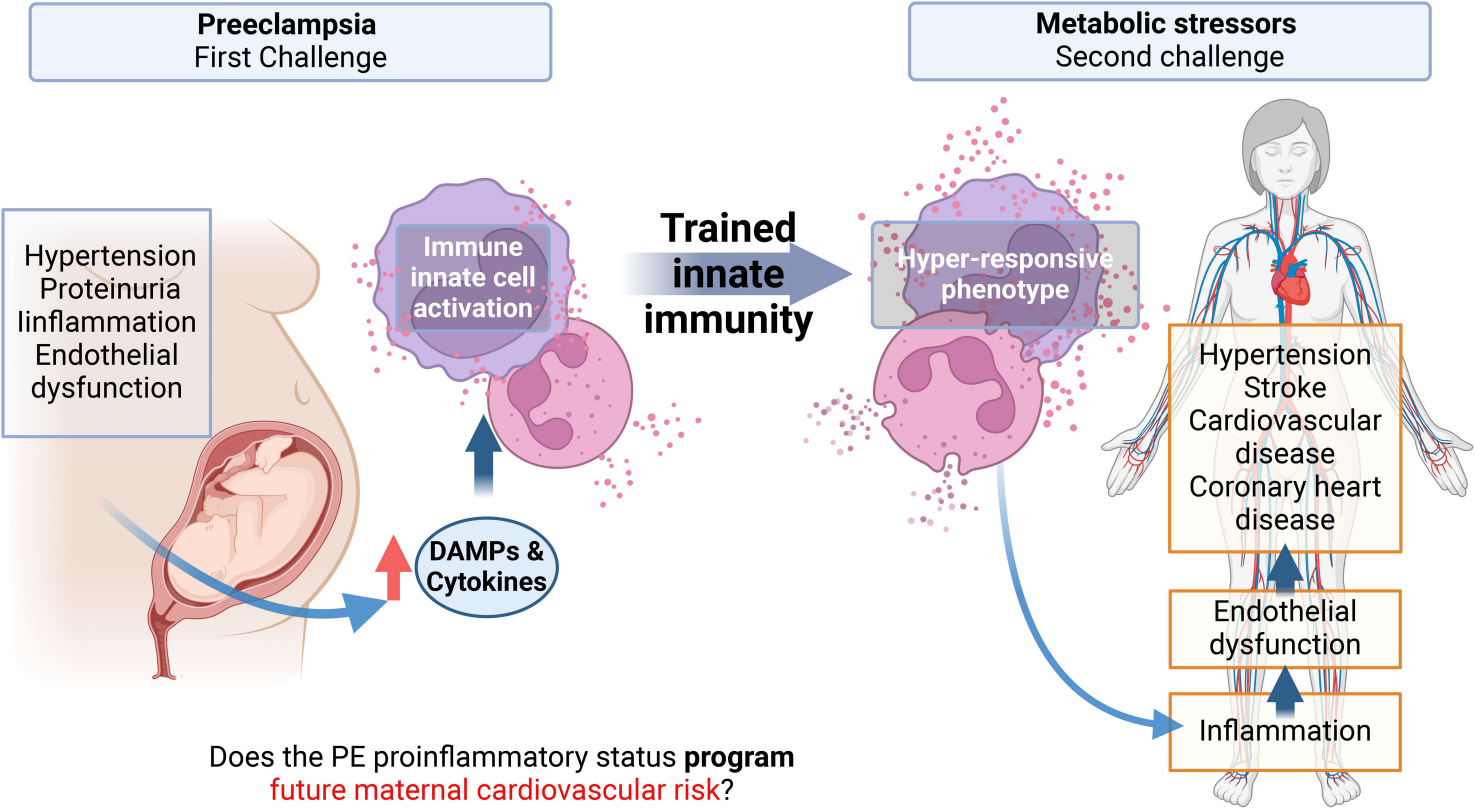
Graphical abstract. Preeclampsia is associated with an increased cardiovascular risk in the mother, observed months to years after the syndrome with no possible cure proposed yet. A characteristic of preeclampsia is a maternal systemic proinflammatory status with, among others, elevated levels of circulating cytokines and damage-associated molecular patterns (DAMPs). These molecules can activate innate immune cells and endothelial cells, inducing endothelial dysfunction, which is the basis of hypertension, the pathognomonic sign of Preeclampsia. Monocytes and neutrophils activated by DAMPs can result in a phenomenon called trained innate immunity (TRIM) by epigenetic mechanisms, characterized by a hyper-responsiveness of these cells to a second heterologous challenge. This memory can be maintained in myeloid precursors, for several cell generations. This project proposes that the proinflammatory state of EOPE can induce TRIM in monocytes and neutrophils during pregnancy. Then, in the maternal future, these trained monocytes will be hyper-responsive against new molecules associated with metabolic risk factors. This hyper-responsive phenotype could then activate endothelial cells generating endothelial dysfunction in the mother, favoring the appearance of cardiovascular disorders.

TRIM is associated with cardiovascular disorders (105), making it possible that PE-induced long-term TRIM could impact endothelial homeostasis. Then, as shows the **Figure 1**, we proposed the proinflammatory status of preeclampsia constituted at least by high concentration of pro-inflammatory cytokines and increased levels of several DAMPs (compiled in **Table 1**) is associated with the activation of innate cells, including monocytes and neutrophils. This context, as was discussed above, could be conducive to TRIM acquisition during the syndrome. Then, in a short-medium or long-term, the maternal trained innate cells could over respond to new challenges and generate a strong and fast proinflammatory status disturbing the cardiovascular physiology of women (see **Figure 1**). Regarding the moment during the pregnancy at which DAMPs could initiate in PE the challenge in innate immune cells is not clear. However, DAMPs seem to have permanent participation in the pathophysiology of the syndrome.

**Table 1:** Maternal circulatory DAMPs with increased levels in Preeclampsia.

DAMP	Level in control and preeclamptic pregnancy	Ref.	Physiological sub-cellular distribution	Physiological function	Receptor that sense DAMP
Cell-free fetal DNA (cffDNA)	Control: 46.9 [20.8-78.2]	(106)	Nucleus	Source of all intrinsic genetic information (107)	TLR-9 (108)
	PE: 594.8 [240-1090.4]				
	GE/mL. Median [IQR]				
Cell-free mitochondrial DNA (cfmtDNA)	Control: 239.3 [197.4-297.1]	(109)	Mitochondrial matrix	Encodes 13 crucial proteins that are part of the oxidative phosphorylation system (110)	TLR-9 (111)
	PE: 271.5 [220.2-335.1]				
	Copy number. Median [IQR]				
Cell-free heme (cfHeme)	Control: 1.63 ± 0.22	(112)	Cytosol	Component of several proteins contributes to vasodilation, cellular signaling, iron balance, and provides antioxidant and cellular protection (113)	TLR-4 (114)
	PE: 3.18 ± 0.35				
	µM. Mean ± SD				
Cyclophilin A (CypA)	Control: 8.71 (5.03–54.12)	(115)	cytosol	Crucial for protein folding, modulates immune response by activating T cells and producing cytokines (116)	CD147 (integrin β2) (117)
	PE: 48.35 (8.12–58.91)				
	ng/mL. Median (min–max)				
Heat Shock Protein 70 (HSP70)	Control: 643.4 [12.7–1084.9]	(78)	Cytosol, nucleus, ER, and mitochondria	Assists in the correct protein folding, prevents aggregation, participates in the degradation of damaged proteins (118)	TLR-2 and TLR-4 (119)
	PE: 901.1 [401.6–1263.8]				
	pg/mL. Median [IQR]				
High Mobility Group Box 1 (HMGB1)	Control: 2.1 [1.5–4.7]	(120)	Nucleus	Organizes DNA and nucleosomes in the nucleus, facilitating gene transcription (121)	RAGE and TLR-4 (76)
	PE: 5.5 [2.3–78.1]				
	ng/mL. Median [IQR]				
Histones (Hs)	Control: 1.31 ± 0.54	(122)	Nucleus	Compact DNA into nucleosomes and regulate gene expression through its post-translational modifications (123)	TLR-4 (124) Clec2d (125)
	PE: 4 ± 0.85				
	Fold change. Mean ± SD				
Hyaluronan (HA)	Control: 58.9 [2.5–180.7]	(78)	Extracellular matrix	Provide structural support, and promoting healing by facilitating cell migration and proliferation (126)	CD44 (127)
	PE: 127.5 [20.7–287.6]				
	ng/mL. Median [IQR]				
Myeloid-related protein 8, MRP8 (S100A8) and MRP14 (S100A9). Heterodimer Calprotectin (S100A8/A9)	Control: 552 (471–651)	(128)	Cytosol	Myelomonocytes metal-chelating antimicrobial protein of the innate immune response (129)	TLR-4 (130)
	PE: 1081 (865–1569)				
	µg/L. Median (95% CI)				
Uric Acid (UA)	Control: 4.2 [2.8–4.8]	(78)	Cytosol	Uric acid is the end product of exogenous and endogenous purine metabolism (131)	P2X7 (132)
	PE: 6.1 [4.5–10.1]				
	ng/dL. Median [IQR]				

## DAMPs and the pathophysiology of preeclampsia

There is no clue as to whether DAMPs could be involved in the origin of PE, however it is possible to propose that DAMPs could maintain and even amplify the pro-inflammatory status of the syndrome. Exposure of control placental explants to PE serum increased HMGB1 release (133). Ex vivo induction of oxidative stress and hypoxia to control placenta increased liberation of several DAMPs, including HMGB1, HSP70, S100A8, S100A12 and Cell free-fetal DNA (18). The latter together with the findings that the receptors TLR-2,-3, -4, and -9 are increased in syncytiotrophoblast in PE (134) suggest a self-maintaining of the DAMP-induced proinflammatory status of the placenta.

DAMPs may enter maternal circulation, as several with high maternal plasma levels in PE are listed in **Table 1**, including cell-free DNA, crystals, and proteins. Although most of them can be released by the placenta it is not possible to determine the original source of them. However, DAMPs could induce alteration in endothelium. Thus, microvascular endothelial cells HMEC-1 treated with recombinant HMGB1 elevated the expression of the adhesion molecule ICAM-1 favoring the arrest of the monocyte cell line U937 on them (133). Cell-free mitochondrial DNA (cfmtDNA) also generated a similar effect, in this case cfmtDNA could increase macrovascular endothelial cell EA.hy926 permeability, together with the rising of ICAM and E-selectin expression which favored the arrest of primary polymorphonuclear leukocyte (135). In the other hand, the cfmtDNA released by hypoxic murine trophoblast reduced the endothelial-dependent vasodilation in abdominal aorta, partially mediated by NLRP3 since the effect on cfmtDNA diminished in NLRP3 ^-/-^ animals (136). The latter together with the fact that preeclampsia curses along endothelial dysfunction (137) suggests that elevated circulating DAMPs may be part of the syndrome.

Maternal plasma in PE shows elevated levels of the anti-angiogenic protein soluble fms-like tyrosine kinase 1 (sFLT-1) (138). sFLT-1 is proposed to be one of the responsible for endothelial dysfunction in PE by the sequestration of VEGF resulting in the endothelial expression of the adhesion proteins ICAM and VCAM and the vasoconstrictor peptide endothelin-1 (139). sFLT-1 can be released by THP-1-derived macrophages followed by the activation of inflammasome in a GSDMD dependent manner (140). In this sense the DAMPs hyaluronan and HSP70 (141), and uric Acid (142) generated a strong inflammasome activation in primary monocytes from mothers with PE probably contributing to the high levels of IL-1β and IL-18 found in PE-maternal plasma (142). The activation of inflammasomes also participates in the releasing of DAMPs since the induction of pyroptosis led to the liberation of HMGB1 (143).

In the most severe cases of preeclampsia, mothers have a higher risk of thrombotic events during pregnancy (144). In fact, the plasma from mothers with PE had fast and strong thrombin generation compared to control pregnancy plasma (122). The same study showed that plasma from preeclampsia patients strongly induced NETosis in neutrophils from healthy donors (122). NETosis is a neutrophil-specific activation characterized by the release of neutrophil extracellular traps (NETs), which consist of chromatin and antimicrobial proteins (145). In PE high levels of NETs were found in maternal circulation (146). The link between NETs thrombosis is based on the capacity of human nuclear-DNA and histones for inducing thrombin generation (147). Interestingly, intact-NETs or assembled histones are unable to induce thrombin generation, indicating that NETs must be dissembled to have coagulatory activity. Based on the latter, DAMPs may favor the elevated risk of thrombotic events in mothers-with-PE due to the high levels of circulatory histones (see **Table 1**), and to the induction of NETosis by HMGB1 (148).

**Figure 2:**
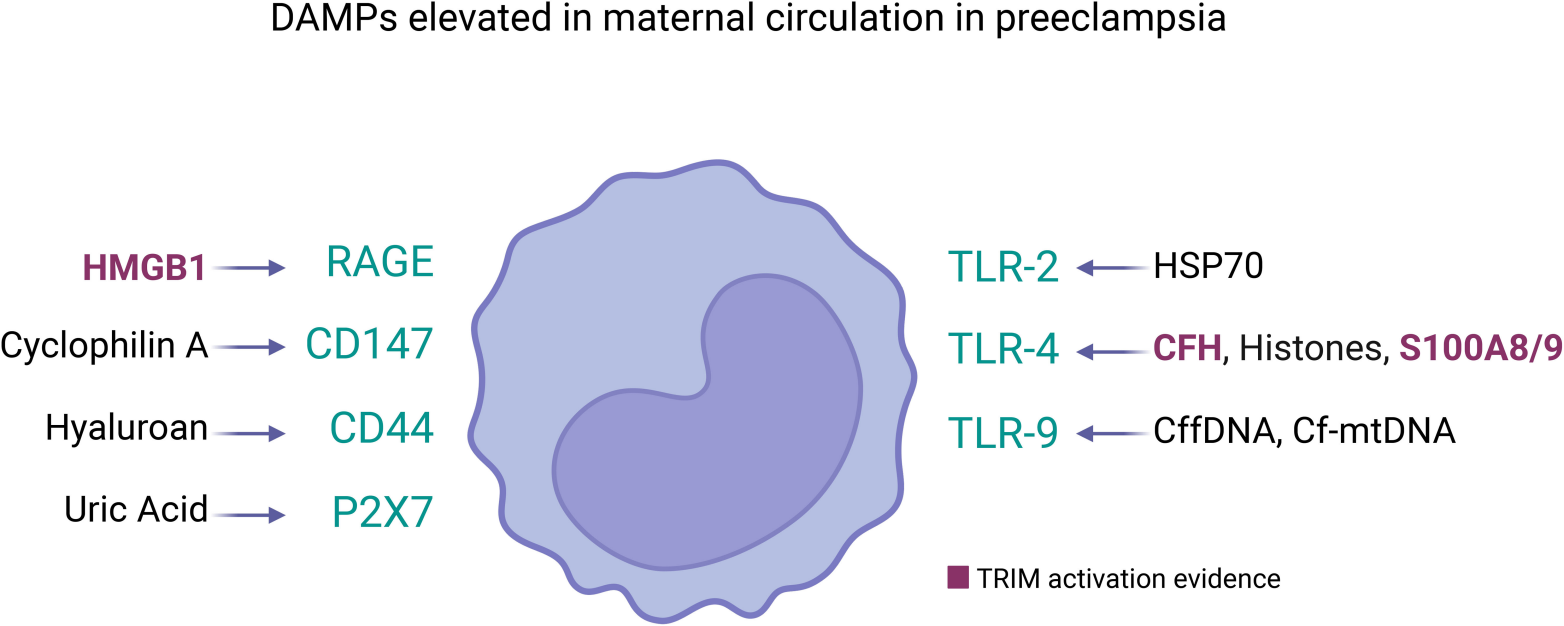
DAMPs elevated in maternal circulation in preeclampsia and its receptors in innate immune cells. Several DAMPs have high plasmatic concentration in mothers with preeclampsia, each of them can be sensed by pattern recognition receptors in cell membrane of innate immune cells (represented as monocyte). The DAMPs/Receptor are: High Mobility Group Box 1 (HMGB1) (120)/RAGE and TLR4 (114); Cyclophilin A/CD147 (117); Hyaluronan (78)/CD44 (127); Uric Acid (UA) (78)/P2X7 (132); Heat Shock Protein 70 (HSP70) (78)/TLR2 and TLR4 (119); Cell-free heme (CFH) (112)/TLR-4 (149); Histones (122)/TLR-4 (124) and Clec2d (125); Calprotectin (S100A8/A9) (128)/TLR-4 (123); Cell-free fetal DNA (cffDNA) (106)/TLR-9 (108); Cell-free mitochondrial DNA (cfmtDNA) (109)/TLR-9 (150). Among them, only HMBG1 (75), cell-free heme (151), and S100A8/9 (152) have been showed as TRIM inducers. However, the circulatory DAMPs in PE may act collectively to promote TRIM acquisition in innate immune cells during the syndrome.

In the context of PE, as **Table 1** and **Figure 2** show, several DAMPs have increased maternal circulatory concentration in PE, suggesting that TRIM could be induced in innate cells during the syndrome. The latter is supported by the evidenced ability of cell-free heme (151) and HMGB1 (75) to induce TRIM. In the case of S100A8/A9, the evidence indicates a possible dual role as pro-inflammatory molecules (153) but also as an immune modulator (152). The latter indicates that it will be necessary to evaluate not only if individual DAMPs with high levels in PE can induce TRIM but also how collectively high levels of different DAMPs affect TRIM acquisition.

## TRIM on myeloid progenitors

The fact that mature innate immune cells are short-lived (*i.e.* Half-life 5-7 days (154, 155)), raise the unanswered question about how PE-induced TRIM could last years. In this scenario, it would be necessary that the precursors of innate cells also be involved. In this sense, human hematopoietic stem and progenitors cells (HSPCs) showed permanent alterations after *in vivo* Bacille Calmette-Guérin (BCG) vaccination (150), a classic TRIM inducer (156). The latter included, a permanent (i.e. at least 90 days post-vaccination) transcriptional reprogramming in HSPCs, leading to an upregulation of genes associated with myeloid and granulocytic cell lineage priming, generating a myeloid differentiation bias within HSPCs, and enhanced proinflammatory response to various stimuli of mature peripheral blood mononuclear cells (150). Regarding DAMPs, TRIM induced by heme in mice showed an increase in myeloid-biased long-term hematopoietic stem cells and multipotent progenitors with an expansion of myeloid-biased, associated to elevated chromatin accessibility in genes associated with myeloid differentiation of HSPCs, also there was a significant and permanent increase in mature myeloid cells (i.e. neutrophils and monocytes), with an enhanced phagocytic activity (151). HSPCs express TLR-2, - 4, and -9, and their activation induces the differentiation and expansion to macrophages (157) suggesting that DAMPs may reach bone marrow and promote TRIM-associated permanent modifications.

## Conclusions

Preeclampsia is a severe multisystemic syndrome which manifest with different pathological characteristics (*i.e.* EOPE and LOPE). Additionally, it remains unclear what are the mechanisms that induce the syndrome. However, one fact is clear: mothers who had PE were at higher cardiovascular risk. Thus, mothers not only face a serious pregnancy pathology, but this syndrome will probably also affect their future health. Therefore, an understanding of the mechanisms that underlie higher cardiovascular risk is crucial. Trained innate immunity has recently changed the paradigm that adjudicated the immune memory only to T/B cells from adaptive immunity, and this type of epigenetic memory is a mechanism with a clear potential to impact cardiovascular physiology. This epigenetic memory could explain the increased cardiovascular risk observed in women who have experienced PE, potentially triggered by future health challenges throughout their lives. However, further research is required to explore this hypothesis, as no study has directly examined this possibility to date. If PE-related sterile inflammation can induce TRIM, testing seems to be mandatory since several research groups are focused on TRIM modulation (149). Thus, this offers a certain possibility to improve the future maternal health of women who have preeclampsia.
